# Two *Candida auris* Cases in Germany with No Recent Contact to Foreign Healthcare—Epidemiological and Microbiological Investigations

**DOI:** 10.3390/jof7050380

**Published:** 2021-05-12

**Authors:** Joerg Steinmann, Thomas Schrauzer, Lisa Kirchhoff, Jacques F. Meis, Peter-Michael Rath

**Affiliations:** 1 Institute of Clinical Hygiene, Medical Microbiology and Infectiology, Klinikum Nürnberg, Paracelsus Medical University, 90419 Nuremberg, Germany; thomas.schrauzer@klinikum-nuernberg.de; 2 Institute of Medical Microbiology, University Hospital Essen, University of Duisburg-Essen, 45147 Essen, Germany; lisa.kirchhoff@uk-essen.de (L.K.); peter-michael.rath@uk-essen.de (P.-M.R.); 3 Department of Medical Microbiology and Infectious Diseases, Canisius-Wilhelmina Hospital, 6532 SZ Nijmegen, The Netherlands; jacques.meis@gmail.com; 4 Centre of Expertise in Mycology Radboudumc, Canisius-Wilhelmina Hospital, 6532 SZ Nijmegen, The Netherlands

**Keywords:** *Candida auris*, resistance, STR typing, infection control

## Abstract

*Candida auris* has become a global fungal public health threat. This multidrug-resistant yeast is associated with nosocomial intra- and interhospital transmissions causing healthcare-associated infections. Here, we report on two *C. auris* cases from Germany. The two patients stayed in Germany for a long time before *C. auris* was detected during their hospitalization. The patients were isolated in single rooms with contact precautions. No nosocomial transmissions were detected within the hospital. Both *C. auris* isolates exhibited high minimum inhibitory concentrations (MICs) of fluconazole and one isolate additionally high MICs against the echinocandins. Microsatellite genotyping showed that both strains belong to the South Asian clade. These two cases are examples for appropriate in-hospital care and infection control without further nosocomial spread. Awareness for this emerging, multidrug-resistant pathogen is justified and systematic surveillance in European health care facilities should be performed.

## 1. Introduction

*Candida auris* has become a worldwide public health threat [1]. This fungus is associated with multidrug resistance and a high mortality. Within a hospital *C. auris* can survive on inanimate surfaces for long periods and nosocomial transmissions were repeatedly reported from all over the world [2]. Compared to Asia, USA and South Africa the *C. auris* incidence in Europe is, despite outbreaks reported in Spain and UK [3,4], low. Between January 2018 and May 2019, 349 cases were reported in the European Union/European Economic Area [5]. In Germany only a few *C. auris* cases have been reported so far. In a series of seven German patients, six of these cases were previously hospitalized abroad or had contact to foreign healthcare [6]. Colonization with *C. auris* has been detected at various body sites, including nares, groin, axilla, ears and rectum [7]. Risk factors for colonization are among others elderly age, diabetes, indwelling medical devices, immunosuppression, hemodialysis/chronic renal disease and use of broad-spectrum antibiotics and/or antifungals [8]. Receiving carbapenems and systemic fluconazole in the prior 90 days, being on a ventilator and having ≥ 1 acute care hospital visit in the prior six months were shown to be associated with *C. auris* colonization in New York nursing facilities with ventilator units [9]. Furthermore, contact with *C. auris* positive patients or their environment is an additional risk factor in healthcare institutions. Recently, *C. auris* emerged in an Italian hospital in context with the COVID-19 pandemic [10]. In most of the European hospitals no clear screening strategies for *C. auris* are established [11]. However, UK guidelines recommend that all hospitals should establish a *C. auris* screening policy after local risk assessment of those patients most likely to be colonized [12]. In Germany, all admitted patients with a history of hospitalization in a country with a high prevalence of multidrug-resistant (MDR) gram-negative bacteria should be screened for MDR bacteria and placed in contact precautions in a private room [11]. In contrast, similar recommendations to screen for *C. auris* in Germany do not exist so far.

Here we present the epidemiological, clinical, and mycological details of two *C. auris* cases in Germany highlighting that the time point of fungal pathogen acquisition remains open and infection control standards may prevent further transmission.

## 2. Methods and Results

### 2.1. Laboratory Investigations

*C. auris* screening samples from patients and environmental swabs were cultured on chromogenic yeast medium (chromID, bioMèrieux, Nürtingen, Germany or Candida Brilliance, Thermo Fisher Scientific, Wesel, Germany) for 48 h at 37 °C. Identification of *C. auris* was done with MALDI TOF MS (Bruker, Bremen, Germany or VITEK MS, bioMèrieux, Marcy-l’Étoile, France) and confirmed by ITS sequencing as described previously [13]. Susceptibility testing was performed by broth microdilution according to CLSI, following M27-S4 [14] and one isolate was tested at the National Reference Centre for Invasive Fungal Infections, Leibniz Institute for Natural Product Research and Infection Biology—Hans Knöll Institute, Jena, Germany according to EUCAST. One *C. auris* isolate was selected for *ERG11* and *FKS1* gene amplification and sequencing [15]. Genotyping was performed by a recently developed short tandem repeat typing method [16].

### 2.2. Cases

**Figure 1:**
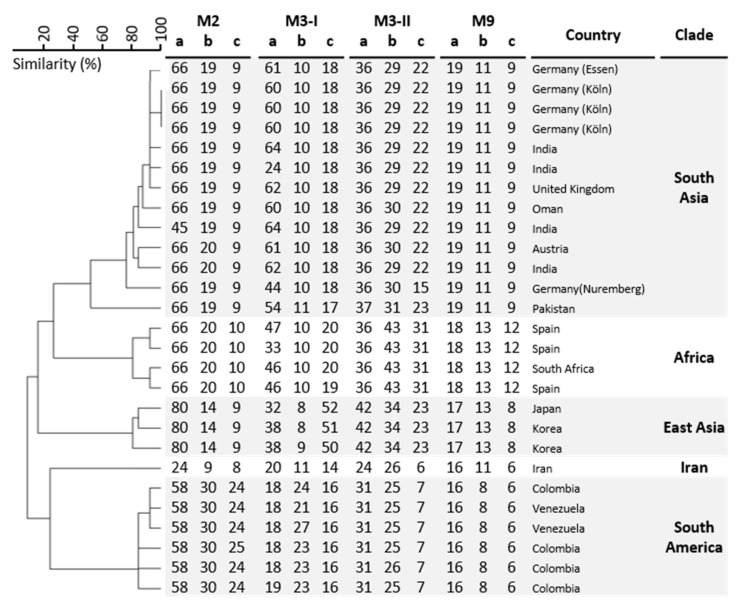
STR genotypes of 27 *C. auris* isolates. In UPGMA dendrogram of STR genotypes, both German isolates (Essen and Nuremberg) are included.

Case 1: A middle-aged male patient with tetraparesis after fracture of the cervical vertebrae 4/5 in Iraq in 2015 came for further treatment to South Germany in 2018 and lived in a long-term care facility since 2019. In 2019 and 2020 the patient had several hospitalizations in Nuremberg, Germany because of pneumonia and recurrent urinary tract infections. He had an indwelling urinary catheter, decubitus ulcer and received several courses of broad-spectrum antibiotics. During his first hospital stay in February 2019, no *C. auris* was detected in screening swabs of rectum, groin and nose. In August 2019, *C. auris* was found for the first time in wound and urine screening samples at admission. During hospital stays in December 2019, April 2020 and September 2020, *C. auris* was found at several body sites including the ears. After *C. auris* was detected in August 2019, the patient was cared for in a single patient room throughout his hospitalizations. Based on results of clinical specimens there was no evidence of transmission of *C. auris* to other patients within the hospital. After patient discharge in April 2020 and subsequent cleaning of the patient room, inanimate surfaces close to the patient environment, e.g., bedrails, were examined for *C. auris* by taking a total of 20 swabs. Examining these swabs revealed no presence of yeasts. The patient never received antifungal treatment during hospitalizations. Susceptibility testing and microsatellite typing results are shown in Table 1 and Figure 1. The strain represents a new (sub)genotype within the South Asian clade.

Case 2: A middle-aged male patient underwent lung transplantation in a tertiary hospital in Essen, Germany. Bronchial cultures of donor and recipient showed no fungal growth. After transplantation, antifungal prophylaxis with voriconazole was started. Regularly performed therapeutic drug monitoring showed trough levels of voriconazole within the therapeutic range (1–4 mg/L). The patient was nursed in a single patient room during the whole stay in the hospital due to colonization with MDR pathogens (*Klebsiella pneumoniae* with extended spectrum beta-lactamase phenotype and resistance to quinolones, vancomycin-resistant *Enterococcus faecium*, carbapenem-resistant *Pseudomonas aeruginosa*). Post-transplant the patient suffered from CMV pneumonia and recurrent septic episodes with *Pseudomonas* and *Klebsiella* as well as episodes of multi-organ failure. While routine microbiology tracheal aspirate samples showed colonization of bronchi with *Candida albicans*, urine samples showed no growth of pathogens. The patient died of cardiogenic-septic shock after a six week stay on an intensive care unit on day 290 after transplantation. He was not discharged home since transplantation.

Five days before his death, yeasts were found in a urine culture without leucocyturia. At this time, only *C. albicans* and *C. glabrata* were cultured from screening samples from the respiratory tract and stool. Blood cultures always remained negative for fungi. The urine culture grew *C. auris.* Broth microdilution susceptibility testing showed that the *C. auris* isolate was resistant to fluconazole (>64 mg/L) and voriconazole (16 mg/L) (Table 1) [17]. The echinocandins micafungin and anidulafungin had an MIC of 2 mg/L. By sequencing the *ERG11*, an amino acid substitution was found at position 132 (Y132F). Further, the isolate was wild type at position 639 in the *FKS1* gene. Immediately after identification of *C. auris*, all patients of the intensive care ward (*n* = 15) were assessed for *C. auris* (respiratory samples, stool, urine), but no further isolates were detected. Furthermore, *C. auris* was not found in routine screening or diagnostic samples of other patients on the wards where the patient stayed before. Genetically, the isolate belongs to the South Asian clade (Figure 1). The patient did not travel abroad for the last years and no other patients with *C. auris* could be identified, therefore the origin of this isolate remains elusive.

## 3. Discussion

This report shows epidemiological and microbiological details of two *C. auris* cases from two distinct German tertiary hospitals without a recent exposure to foreign healthcare. *C. auris* is rarely detected in Germany and other European countries and was so far mainly introduced from patients with contact to healthcare systems from countries with higher prevalence [11,18,19,20]. Both patients had several risk factors for *C. auris* colonization. Epidemiological, phenotypical, and genotyping data showed that there was no link between the patients and/or isolates, indicating two different sources. Because there were no additional infected or colonized cases with *C. auris* identified and environmental sampling was negative, contact isolation precautions and appropriate infection control seemed to prevent further transmissions.

Even though no *C. auris* case from Iraq was reported, it is possible that the first patient was already colonized with *C. auris* in Iraq years ago, but the fungus was never detected during previous hospital stays in Germany. *C. auris* may not have been detected on screening because the fungal load may have been below the limit of detection. A patient-to-patient transmission as the potential source of *C. auris* colonization in the long-term care facility in Germany cannot be excluded as the residents were not screened for *C. auris*. It has been shown earlier that *C. auris* can be highly prevalent in residents and in the environment of long-term care facilities in the USA [9].

Both patients were each nursed in single patient rooms during their hospital stay. Beyond basic hygiene, isolating colonized or infected patients with contact precautions, and regularly cleaning/decontaminating the environment is helpful to prevent further transmission [21,22]. The first patient was colonized with *C. auris* at several body sites, but no room contamination at high touch, inanimate surfaces could be found after terminal room cleaning. In addition, patient contact screening of direct contacts including those being discharged from the health care facility should be performed [22].

An open question remains whether both patients were already colonized with *C. auris* at hospital admission and detection was only possible due to selection factors (e.g., anti-infective therapy) during stay. There is only limited data from European hospitals on the prevalence of *C. auris* colonization at admission. In a study from London the prevalence of *C. auris* in admitted patients to the hospital was 0.04% (*N* = 1/2246) [3]. In a recent study from England, 921 patients from 998 admissions to eight ICUs in three major cities in 2017 to 2018 were screened for *C. auris* at admission [12]. No *C. auris* isolate was detected. It was concluded that widespread screening for *C. auris* in ICUs in England is not recommended and unlikely to be cost-effective. An admission screening strategy with focus on high-risk individuals based on local risk assessment was suggested [12]. Based on the current literature and our experience, a screening strategy for *C. auris* in patients with contact to foreign healthcare and with multiple risk factors including regional factors (e.g., outbreak in the area) can be considered in low prevalence countries. It is currently unclear, whether only an admission screening or additional repeated screening for *C. auris* should be conducted. A case-by-case decision may also be appropriate.

One isolate from Essen had elevated anidulafungin and micafungin MICs, but the patient never received antifungal therapy with echinocandins within the hospital. He received long treatment with voriconazole and the isolate exhibited high MICs against this tri-azole and also to fluconazole. In the USA, about 90% of *C. auris* isolates have been found to be resistant to fluconazole, about 30% resistant to amphotericin B, and less than 5% resistant to echinocandins [23]. We found an amino acid substitution in *ERG11* at position 132 (Y132F). This substitution has been found previously in azole-resistant *C. auris* isolates. [15,24]. Additionally, the isolate was wildtype at position S639 in the *FKS1* gene. A large study from Austria failed to find *FKS1* mutations in *C. albicans* isolates with borderline echinocandin MICs [25]. The fact that our *C. auris* isolate had no mutation in the *FKS1* region is congruent to observations that a mutation is mainly linked to high MICs above 4 mg/L [15]. EUCAST did not provide clinical breakpoints so far. The CDC reported tentative breakpoints for caspofungin with MICs of ≥2 mg/L as well as anidulafungin and micafungin of ≥4 mg/L [26]. A comparison of EUCAST and CLSI reference microdilution showed that six *C. auris* isolates, with elevated echinocandin MICs (4 mg/L), had the same results as measured by both methods for micafungin and anidulafungin (4.9%) [27].

Four major clades of *C*. *auris* (I, II, III, and IV initially originating from South Asia, East Asia, South America, and South Africa, respectively) [26] and a fifth clade reported from Iran have been described to date [28]. Our isolates were from clade I as shown by short tandem repeat typing, a method which is comparable with whole-genome sequencing [7,16]. The two described isolates differ in three markers indicating that they are not related.

This case report has some limitations. The two reported cases were retrospectively investigated and not all screening samples were performed according to the recommendations. Furthermore, for both patients the time point of acquisition of *C. auris* cannot be definitely determined and it cannot be excluded that an undetected hospital transmission may have occurred.

These two potentially “endemic” German *C. auris* cases show the importance of awareness of *C. auris* in hospitals in Europe not only for patients with contact to foreign healthcare environments. European health care facilities should establish local guidelines for *C. auris* screening for patients with risk factors and implement strict infection control measures for positive cases.

## Figures and Tables

**Table 1:** In vitro antifungal activities of the two *C. auris* isolates from Germany in mg/L.

	AMB	FZ	VRC	POS	ISA	MCF	ANF
Isolate 1	1	64	1	≤0.016	≤0.016	nd	0.25
Isolate 2	2	>64	16	0.25	8	2	2

AMB, amphotericin B; FZ, fluconazole; VRC, voriconazole POS, posaconazole; ISA, isavuconazole; MCF, micafungin; ANF, anidulafungin; nd, not determined.

## Data Availability

Data are available within the article.
